# *Orthosiphon stamineus* Proteins Alleviate Pentylenetetrazol-Induced Seizures in Zebrafish

**DOI:** 10.3390/biomedicines8070191

**Published:** 2020-07-02

**Authors:** Yin-Sir Chung, Brandon Kar Meng Choo, Pervaiz Khalid Ahmed, Iekhsan Othman, Mohd. Farooq Shaikh

**Affiliations:** 1 Neuropharmacology Research Laboratory, Jeffrey Cheah School of Medicine and Health Sciences, Monash University Malaysia, Bandar Sunway 47500, Malaysia; chung.yinsir@monash.edu (Y.-S.C.); Brandon.Choo@monash.edu (B.K.M.C.); Iekhsan.Othman@monash.edu (I.O.); 2 Liquid Chromatography-Mass Spectrometry (LCMS) Platform, Jeffrey Cheah School of Medicine and Health Sciences, Monash University Malaysia, Bandar Sunway 47500, Malaysia; 3 School of Business, Monash University Malaysia, Bandar Sunway 47500, Malaysia; pervaiz.ahmed@monash.edu; 4 Global Asia in the 21st Century (GA21), Monash University Malaysia, Bandar Sunway 47500, Malaysia

**Keywords:** *Orthosiphon stamineus*, plant-derived proteins, epilepsy, seizures, zebrafish

## Abstract

The anticonvulsive potential of proteins extracted from *Orthosiphon stamineus* leaves (OSLP) has never been elucidated in zebrafish (*Danio rerio*). This study thus aims to elucidate the anticonvulsive potential of OSLP in pentylenetetrazol (PTZ)-induced seizure model. Physical changes (seizure score and seizure onset time, behavior, locomotor) and neurotransmitter analysis were elucidated to assess the pharmacological activity. The protective mechanism of OSLP on brain was also studied using mass spectrometry-based label-free proteomic quantification (LFQ) and bioinformatics. OSLP was found to be safe up to 800 µg/kg and pre-treatment with OSLP (800 µg/kg, i.p., 30 min) decreased the frequency of convulsive activities (lower seizure score and prolonged seizure onset time), improved locomotor behaviors (reduced erratic swimming movements and bottom-dwelling habit), and lowered the excitatory neurotransmitter (glutamate). Pre-treatment with OSLP increased protein Complexin 2 (Cplx 2) expression in the zebrafish brain. Cplx2 is an important regulator in the trans-SNARE complex which is required during the vesicle priming phase in the calcium-dependent synaptic vesicle exocytosis. Findings in this study collectively suggests that OSLP could be regulating the release of neurotransmitters via calcium-dependent synaptic vesicle exocytosis mediated by the “Synaptic Vesicle Cycle” pathway. OSLP’s anticonvulsive actions could be acting differently from diazepam (DZP) and with that, it might not produce the similar cognitive insults such as DZP.

## 1. Introduction

Epilepsy is a chronic non-communicable disease of the brain that affects around 70 million people of all ages worldwide and accounts for about 1% of the global burden of disease. Epilepsy has a high prevalence and an estimated five million people are diagnosed with epilepsy each year. Epilepsy is characterized by recurrent seizures due to brief disturbances in the electrical functions of the brain. It involves brief episodes of involuntary movement that lead to changes in sensory perception, motor control, behavior, autonomic function, or sometimes loss of consciousness [1]. To date, despite having more than 30 antiepileptic drugs (AEDs) on the market [2,3], there are still difficulties in reaching the goal of treating epilepsy and its associated complications without adverse effects. Globally, epilepsy remains a public health imperative.

People with epilepsy often require lifelong treatment. AEDs are the mainstay of treatment. These conventional drugs bring about clinically worthwhile improvements but have tolerability issues due to their side effects. Many AEDs used in current mainstream clinical practice have been reported to elicit undesired neuropsychological consequences such as depression (24% lifetime prevalence), anxiety (22%), and intellectual disability, particularly in children with epilepsy (30%–40%) [1]. More than one-third of epileptic seizures are not well controlled by a single AED and often require treatment with two or more AEDs (add-on therapy) [1,2]. Furthermore, about 40%–60% of epileptic patients, accounting for both children and adults, develop neuropsychological impairments [3]. This drives a significant portion of epileptic patients to seek alternative interventions, particularly in herbal medicine [4]. Current systematic studies are reporting promising anticonvulsive activities in a constellation of medicinal plants [5,6].

*Orthosiphon stamineus* (OS) or *Orthosiphon aristatus var. aristatus* (OAA), also commonly known as cat’s whiskers or “misai kucing,” is an important medicinal plant. Choo et al. (2018) has shown that the ethanolic extract of OS, exhibited anticonvulsive activity in zebrafish Choo, Kundap [7] and Coelho et al. (2015) has demonstrated the anticonvulsant potential of rosmarinic acid in mice, which is an active chemical constituent in OS extract Coelho, Vieira [8]. Nonetheless, until now the protective potential of OS primary metabolites has not been studied, let alone its proteins. The proteins extracted from OS leaves (OSLP) may also hold valuable protective potential for central nervous system (CNS) disorders such as epilepsy. In the research of epilepsy and drug discovery, zebrafish (*Danio rerio*) has been widely recognised as an important and promising vertebrate model. Genetic profile of zebrafish shares approximately 70% similarity with human and about 84% of genes known to human diseases are also expressed in zebrafish [9,10]. This makes the zebrafish model particularly useful as a high-throughput screening system in studying mechanisms of brain functions and dysfunctions [11]. To the best of our knowledge, this is the first study on elucidating the anticonvulsive potential of proteins extracted from OSLP.

## 2. Experimental Section

### 2.1. Materials Chemicals and Apparatuses

L-Glutamic acid (Glu), Gamma-Aminobutyric acid (γ-aminobutyric acid), Pentylenetetrazol (PTZ), Diazepam (DZP), Benzocaine, complete EDTA-free protease inhibitors, phosphatase inhibitors cocktail 2, dithiothreitol (DTT), trifluoroethanol (TFE), ammonium bicarbonate (ABC), 2,3,5-triphenyltetrazolium chloride (TTC), formic acid (FA), and methanol (MeOH) of HPLC-grade were purchased from Sigma-Aldrich (St. Louis, MO, USA). Pierce^®^trypsin protease, Pierce^®^ Radioimmunoprecipitation assay (RIPA) buffer of mass spec grade and Pierce^®^C18 mini spin columns were purchased from Thermo Scientific Pierce (Rockford, IL, USA). Protein LoBind microcentrifuge tube (Eppendorf, Enfield, CT, USA), acetonitrile (ACN), trifluoroacetic acid (TFA), indoleacetic acid (IAA) and CHAPS (Nacailai Tesque, Kyoto, Japan) of mass spec grade were from Sigma-Aldrich (St. Louis, MO, USA), Quick Start™ Bradford Protein Assay Kit from Bio-Rad (Hercules, CA, USA), Dimethylsulfoxide (DMSO) and 37% formaldehyde solution were from Friendemann Schmidt Chemical (Parkwood, Western Australia), Milli-Q ultrapure (MQUP) water from Millipore GmbH (Darmstad, Germany), acetic acid (glacial, 100%) from Merck (Darmstadt, Germany) and Phosphate buffered saline (PBS) tablets from VWR Life Science AMRESCO^®^ (Radnor, PA, USA). Liquid nitrogen was purchased from Linde Malaysia, Hamilton syringes 25 µL (MICROLITER™ #702) from Hamilton Co. (Reno, NV, USA), 35 gauge needles (PrecisionGlide™) were from Becton, Dickinson and Company (Franklin Lakes, NJ, USA), ultrasonic cell crusher (JY88-II N, Shanghai Xiwen Biotech. Co., Ltd., Shanghai, China), Eyela SpeedVac Vacuum Concentrator (Thermo Scientific Pierce, Rockford, IL, USA), Camry High-Precision Electronic Pocket Scale (Model EHA901, Zhaoqing, China) and Classic pH Pen Tester from Yi Hu Fish Farm Trading Pte. Ltd. (Singapore). The other chemicals of analytical grade were from established suppliers worldwide.

### 2.2. Software and Equipment

For the behavioral study, SMART V3.0.05 tracking software (Panlab Harvard Apparatus, Barcelona, Spain) was used for the automated tracking of zebrafish swimming patterns. The video recorded using the camcorder was analyzed using the software. The water-filled tank was divided into two halves of the same size; the upper-half was marked as the top zone and the lower-half as the bottom zone as described by Kundap et al. 2017 [12].

For the neurotransmitter analysis, the solvent delivery was performed using Agilent Ultra High-Performance Liquid Chromatography (UHPLC) 1290 Series (Agilent Technologies, Santa Clara, CA, USA) consisting of Agilent 1290 Series High-Performance Autosampler, Agilent 1290 Series Binary Pump and Agilent 1290 Series Thermostatted Column Compartment; the separations were performed using Zorbax Eclipse Plus C18 (Rapid Resolution HD, 2.1 × 150.0 mm with 1.8 µM pore size reverse-phase column) (Agilent Technologies, Santa Clara, CA, USA), and coupled with Agilent 6410B Triple Quadrupole (QQQ) mass spectrometer equipped with an electrospray ionization (ESI) (Agilent Technologies, Santa Clara, CA, USA) to detect the targeted neurotransmitters.

In the protein expression study, Agilent 1200 series HPLC coupled with Agilent 6550 iFunnel Quadrupole Time of Flight (Q-TOF) LC/MS, C-18 300Ǻ Large Capacity Chip (Agilent Technologies, Santa Clara, CA, USA) and Agilent MassHunter data acquisition software were used to identify the differentially expressed proteins (Agilent Technologies, Santa Clara, CA, USA). In addition, PEAKS^®^Studio software (Version 8.0, Bioinformatics Solution, Waterloo, ON, Canada) and UniProtKB (Organism: *Danio rerio*) database were used for the analysis of mass spectrometry-based label-free proteomic quantification (LFQ). Cytoscape software (Version 3.7.2 plugin BiNGO for Gene Ontology (GO) annotated information, Cytoscape Consortium, San Diego, CA, USA), Zebrafish Information Network (ZFIN) Database Information, KAAS (KEGG Automatic Annotation Server Version 2.1, Kanehisa Lab., Kyoto, Japan) and KEGG PATHWAY Database (Organism: *Danio rerio*) were used to study the functional annotations, protein-protein interactions, and systemic pathway enrichment analysis.

### 2.3. Zebrafish Maintenance and Housing Conditions

Adult zebrafish (*Danio rerio*; 3–4 months old) of heterogeneous strain wild-type stock (standard short-fin phenotype) were housed in the Animal Facility of Monash University Malaysia and maintained under standard husbandry conditions as follows: standard zebrafish tanks (length of 36 cm × width of 22 cm × height of 26 cm) equipped with circulating water systems to provide constant aeration, controlled water temperature between 26–28 °C and controlled water pH between 6.8–7.1. They were kept in stress-free and hygienic conditions. The zebrafish aquarium was maintained under a 250-lux light intensity with a cycle of 14-h of light to 10-h of darkness controlled by autotimer (light on at 0800 and light off at 2200). Group housing was practiced (10–12 fish per tank) with the females and males separated. The adult zebrafish were fed ad libitum three times a day (TetraMin^®^ Tropical Flakes) and were supplemented with live brine shrimps (Artemia) purchased from Bio-Marine (Aquafauna Inc., Hawthorne, CA, USA). The adult zebrafish were allowed to acclimatize for a period of seven days to reduce stress before commencing the experiments. The Monash University Malaysia Animal Ethics Committee approved all the animal experimental procedures on 17 January 2019.

### 2.4. Experimental Design

#### 2.4.1. OSLP Safety Study in Adult Zebrafish

A limit test was first performed based on a modified version of the OECD Guidelines for the Testing of Chemicals No. 203 [11,12] and the protocols of Choo et al. [10,13]. Prior to the experimental procedures, all the adult zebrafish were fasted for 24 h. Meanwhile, OSLP powder was completely dissolved in tank water (26–28 °C) and concentrations ranging from 50–1600 µg/kg of zebrafish body weight were freshly prepared. Three-month-old adult zebrafish with an average weight of 0.45–0.50 g were selected. The zebrafish were then divided into 7 groups (Table 1), with 8 fish per group (*n* = 8) as follows:

A clean observation tank was first set up and filled with 13 L of tank water (Milli-Q filtered water used for keeping the zebrafish; 26–28 °C). One zebrafish from the vehicle control (VC) group was then placed in the observation tank and its behavior was recorded for 10 min using a digital camera (Sony, Japan). After finishing recording, the zebrafish was transferred into a clean individual 1 L tank filled with the same water. This procedure was then repeated for all the other zebrafish in the VC group. For the OSLP-treated groups (II–VII), different concentrations of OSLP were injected intraperitoneally (i.p.) into the zebrafish. Before each IP injection, a zebrafish was individually immersed in anesthesia solution (30 mg/L of Benzocaine) until the cessation of movement [10,13,14]. Immediately, the zebrafish was extracted out to determine the body weight and to calculate the injection volume. The injection volume was calculated at a volume corresponding to 10 microliters per gram of body weight (modified from 15). After injection, the zebrafish was immediately transferred back to the 13 L observation tank. Then, the same recording and tank transfer procedure was repeated, as performed in the VC group. All 56 zebrafish were then kept for 96 h in their respective 1 L tanks. They were checked on every 15 min for the first two hours of exposure and every half an hour thereafter for the first day. On subsequent days, the zebrafish were checked on the morning, afternoon, and evening (3 times per day). Any zebrafish found to exhibit signs of pain, suffering, or anomaly according to our predefined monitoring sheet at any checkpoint were humanely euthanized via an overdose of benzocaine. This protocol deviates from the OECD guidelines in that it does not use mortality as the criterion to determine toxic effects due to the concerns of the MARP-Australia in using death as an endpoint.

#### 2.4.2. Anticonvulsive Potential of OSLP in Adult Zebrafish

**Figure 1:**
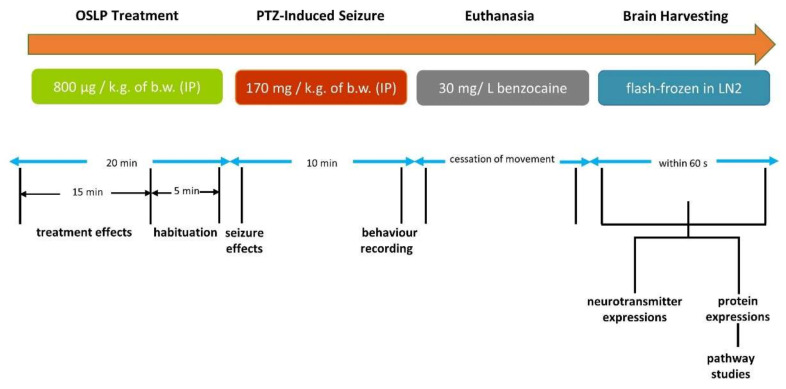
Shows the procedures of experiment.

The anticonvulsive potential of OSLP was investigated in the pentylenetetrazol (PTZ)-induced seizure model. Seizure score and seizure onset time, were one of the primary evaluation parameters used to examine the anticonvulsive activity. Behavioral changes in the zebrafish were determined by evaluating their swimming patterns, total distance travelled (cm) and time spent in the tank (upper-half versus lower-half, s). Three-month-old adult zebrafish with an average weight of 0.45–0.50 g were selected. Prior to beginning the experiments, the zebrafish were kept in 1 L treatment tanks filled with 1 L of tank water (26–28 °C) normally used to fill the zebrafish tanks. In this study, the zebrafish were divided into 5 groups (*n* = 10) (Table 2) and procedures of experiment (Figure 1) were as follows:

All the groups were habituated in their treatment tanks for a half hour before the administration of PTZ. Before each i.p. injection, a zebrafish was individually immersed in anesthesia solution (30 mg/L of Benzocaine) until the cessation of movement. When multiple IP injections were required in tandem on the same zebrafish, the injections were given at alternating lateral ends, rather than the midline between the pelvic fins 10, 13, 14. The VC group was injected with tank water twice. The NC group was first pre-treated with tank water and then PTZ (170 mg/kg) whereas the PC group was pre-treated with diazepam (1.25 mg/kg) followed by PTZ (170 mg/kg). The TC group was injected with 800 μg/kg of OSLP and tank water. The O+P group was pre-treated with OSLP (800 μg/kg) followed by PTZ (170 mg/kg). PTZ-induced seizures lasted for approximately 10 min after the PTZ injection [10,13,14]. All the groups were then transferred to a 13 L observation tank filled three quarters of the way with water. Behavioral changes of the zebrafish were then recorded individually (10 min) with a digital camera (Sony, Japan). The PTZ injected zebrafish presented diverse seizure profiles, intensities and latency in reaching the different seizure scores and seizure onset times. In order to determine the seizure score and seizure onset time, the individual video was analyzed using a computer as per the scoring system below (Table 3) [10,13,14,15,16]:

At the end of the experiment, all the groups were sacrificed. The zebrafish were euthanized with 30 mg/L of Benzocaine until the cessation of movement. The brains were then carefully harvested for neurotransmitter analysis, protein expression study and systemic pathway enrichment analysis.

### 2.5. Extraction of Brains from Zebrafish

At the end of the behavioral studies, the zebrafish brains were carefully harvested from the zebrafish skulls and kept in a sterile Petri dish. Each brain was then immediately transferred into a sterile, pre-chilled 2.0 mL microtube and was flash-frozen in liquid nitrogen (LN_2_) before storing them at −152 °C until further analysis.

### 2.6. Brain Neurotransmitter Analysis Using Nanoflow Liquid Chromatography Coupled with Tandem Mass Spectrometry (Nanoflow-ESI-LC-MS/MS)

The levels of neurotransmitters in the brains, namely gamma-aminobutyric acid (GABA) and glutamate (Glu) were estimated using LC-MS/MS with modifications [13,14,17]. All experiments were performed in 3 independent biological replicates.

A mother stock of neurotransmitter standards was prepared by mixing GABA and Glu in methanol, MQUP water and 0.1% formic acid, to make up a final concentration of 1 mg/mL. Next, serial dilution was performed to prepare 8 points of standard calibrations ranging from 6.25–1000 ng/mL. A blank (methanol, MQUP water in 0.1% formic acid) with a final concentration of 1 mg/mL was also prepared. Together with the 8 points of standard calibrations, they were used for quantifying the levels of GABA and Glu in LC-MS/MS study.

Firstly, each LN_2_ flash-frozen zebrafish brain was homogenized in 1 mL ice-cold methanol/MQUP water (3:1, *vol*/*vol*) using an ultrasonic cell crusher (JY88-II N, Shanghai Xiwen Biotech. Co., Ltd., Shanghai, China). The homogenate was then vortex-mixed (2500 rpm, 3 m) and later incubated on an agitating shaker (4 °C, 1 h). The homogenate was then centrifuged (4 °C, 10,000× *g*, 10 min) and the supernatant was carefully transferred into a sterile 2.0 mL microtube. 100 µL of 0.1% formic acid was slowly added, vortex-mixed (2500 rpm, 3 m) and then centrifuged (4 °C, 10,000× *g*, 10 min). The supernatant was carefully transferred into a sterile insert and vial. Finally, all the brain samples were subjected to LC-MS/MS analysis.

LC-MS/MS was run on an Agilent 1290 Infinity UHPLC coupled with an Agilent 6410B Triple Quad MS/MS equipped with an electrospray ionization (ESI). The separations were performed using Zorbax Eclipse Plus C18 (Rapid Resolution HD, 2.1 × 150.0 mm with 1.8 uM pore size reverse-phase column). The flow rate was 0.3 mL/min with the mobile phase consisting of 0.1% formic acid in water (Solvent A) and acetonitrile (Solvent B). The gradient elution used was: (i) 0 min, 5% Solvent B; (ii) 0–3 min, 50% Solvent B and (iii) 3–5 min, 100% Solvent B, with one-minute post time. The injection volume was 1.0 µL per sample with the column compartment temperature and the autosampler temperature set at 25 °C and 4 °C respectively. The total run time for each injection was 5 min. ESI-MS/MS was used in positive ionization mode with a nitrogen gas temperature of 325 °C, gas flow 9 L/min, nebulizer pressure of 45 psi and the capillary voltage of 4000 V. The MS acquisition was scanned in multiple reaction monitoring (MRM) mode. A calibration range of 1.56–200 ng/mL was used for quantifying the targeted neurotransmitters, with a linear plot where r^2^ > 0.99.

### 2.7. Protein Expression Profiling Using Mass Spectrometry-Based Label-Free Proteomic Quantification (LFQ)

Brains of these two groups, namely NC (injected with PTZ 170 mg/kg) and O+P (pre-treated with OSLP 800 µg/kg followed by PTZ 170 mg/kg) were subjected to tissue lysis to extract the proteins for mass spectrometry-based label-free proteomic quantification (LFQ). All experiments were performed in 4 independent biological replicates.

#### 2.7.1. Protein Extraction from Zebrafish Brain

The zebrafish brain was lysed with 1 mL of ice-cold lysis buffer (RIPA, protease inhibitor 20% *v*/*v*, phosphatase inhibitor 1% *v*/*v*) in a sterile ProtLoBind microtube and then incubated on an orbital shaker (4 °C; 90 min). Next, the content was homogenized using an ultrasonic cell crusher, briefly centrifuged (18,000 × *g*, 4 °C; 10 min) and the supernatant produced was harvested. The supernatant extracted was collected into a new sterile ProtLoBind microtube. Protein concentration was estimated using the Quick Start™ Bradford Protein Assay as instructed by the manufacturer (Bio-Rad, Hercules, CA, USA). After that, the brain lysates were concentrated in a speed-vacuum concentrator (300 rpm; 24 h; 60 °C).

#### 2.7.2. In-Solution Digestion of Proteins

In-solution protein digestion was carried out according to the instructions (Agilent Technologies, Santa Clara, CA, USA). Briefly, protein samples were re-suspended, denatured and reduced in 25 μL of ABC, 25 μL of TFE and 1 μL of DTT, followed by being vortex-mixed (2500 rpm, 3 m) and then heated in an oven (60 °C, 60 min). Next, the samples were alkylated in 4 μL of IAA and were incubated in the dark (60 min, r.t.). After that, 1 μL of DTT was again added to quench excessive IAA (60 min, r.t., in the dark). 300 μL of MQUP water and 100 μL of ABC were added to dilute and adjust the pH of the protein solutions (pH 7–9). Following that, 1 μL of trypsin was added and was then incubated in an oven (37 °C, 18 h, in the dark). Upon completion of incubation, 1 μL of formic acid was added to terminate the tryptic digestion. Finally, all the samples were concentrated in a speed-vacuum concentrator (300 rpm; 24 h; 60 °C, Eyela SpeedVac Vacuum Concentrator). The dry pellets were kept at −20 °C.

#### 2.7.3. De-Salting of Proteins

De-salting of the protein sample was carried out. Each biological replicate was de-salted independently using a Pierce^®^C18 mini spin column as instructed (Thermo Scientific Pierce, Rockford, IL, USA), with modifications. Firstly, each mini spin column was activated in 50% ACN (repeated 3 times, r.t.) and equilibrated in 0.5% of TFA in 5% ACN (repeated 3 times, r.t.). Separately, 90 μL of crude protein was added into 30 μL of sample buffer (2% of TFA in 20%) and briefly vortexed at 2200 rpm to mix well. This step was repeated for all the protein samples. Following that, each of the protein samples was loaded onto a mini spin column and was de-salted (repeated 3 times, r.t.). Subsequently, all the protein samples were washed in 0.5% of TFA in 5% ACN (repeated 3 times, r.t.). Lastly, all the protein samples were eluted in 70% ACN (repeated 3 times, r.t.) and all the flow-through produced was collected, vacuum-concentrated (300 rpm; 24 h; 60 °C) and stored at −20 °C prior to mass spectrometry-based LFQ.

#### 2.7.4. Mass Spectrometry-Based Label-Free Proteomic Quantification (LFQ) Using Nanoflow-ESI-LCMS/MS

De-salted peptides were loaded onto an Agilent C-18 300Ǻ Large Capacity Chip. The column was equilibrated by 0.1% formic acid in water (Solution A) and peptides were eluted with an increasing gradient of 90% acetonitrile in 0.1% formic acid (Solution B) by the following gradient, 3%–50% Solution B from 0–30 min, 50%–95% Solution B from 30–32 min, 95% Solution B from 32–39 min and 95%–3% Solution B from 39–47 min. The polarity of Q-TOF was set at positive, capillary voltage at 2050 V, fragmentor voltage at 300 V, drying gas flow 5 L/min and gas temperature of 300 °C. The intact protein was analyzed in auto MS/MS mode from range 110–3000 *m*/*z* for MS scan and 50–3000 *m*/*z* range for MS/MS scan. The spectrum was analyzed using Agilent MassHunter data acquisition software.

#### 2.7.5. Brain Protein and Peptide Identification by Automated de Novo Sequencing and LFQ Analysis

Protein identification by automated de novo sequencing was performed with PEAKS^®^Studio Version 8.0. UniProtKB (Organism: *Danio rerio*) database (http://www.uniprot.org/proteomes/UP000000437, 46,847 proteins, accessed on 14 February 2020) was used for protein identification and homology search by comparing the de novo sequence tag, with the following settings: both parent mass and precursor mass tolerance was set at 0.1 Da, carbamidomethylation was set as fixed modification with maximum missed cleavage was set at 3, maximum variable post-translational modification was set at 3, trypsin cleavage, the minimum ratio count set to 2, mass error tolerance set as 20.0 ppm and other parameters were set as default by Agilent. False discovery rate (FDR) threshold of 1% and protein score of −10lgP > 20 were applied to filter out inaccurate proteins. PEAKS^®^ indicated that a −10lgP score of greater than 20 is of relatively high in confidence as it targets very few decoy matches above the threshold.

For LFQ analysis, the differentially expressed proteins between the NC (injected with PTZ 170 mg/kg) and O+P (pre-treated with OSLP 800 μg/kg followed by PTZ 170 mg/kg) groups were identified with the following settings: FDR threshold ≤ 1%, fold change ≥ 1, unique peptide ≥ 1, and significance score ≥ 20. PEAKSQ indicated that a significance score of greater than 20 is equivalent to significance *p* value < 0.01. Other parameters were set as default by Agilent.

### 2.8. Bioinformatics Analysis

Bioinformatics analysis (functional annotations, protein-protein interactions and systemic pathway enrichment analysis) of the differentially expressed proteins were analyzed and matched with the databases obtained from GO Consortium, ZFIN (www.zfin.org) and the KEGG PATHWAY Database (*Danio rerio*) [13]. KAAS provides functional annotation of genes by BLAST or GHOST comparisons against the manually curated KEGG GENES database. The result contains KO (KEGG Orthology) assignments (bi-directional best hit) and automatically generated KEGG pathways. The KEGG pathway maps organism-specific pathways: green boxes are hyperlinked to GENES entries by converting K numbers (KO identifiers) to gene identifiers in the reference pathway, indicating the presence of genes in the genome and also the completeness of the pathway.

### 2.9. Statistical Analysis

For behavioral study and neurotransmitter estimation, statistical analysis was performed using GraphPad Prism version 8.0. All data were expressed as mean ± standard error of the mean (SEM). One-way analysis of variance (ANOVA) followed with Dunnett’s post-hoc test at significance levels of * *p* < 0.05, ** *p* < 0.01 and *** *p* < 0.001 against the negative control group (NC, 170 mg/kg PTZ). PEAKSQ statistical analysis (built-in statistical tool of PEAKS^®^ software) was used in the analysis of differentially expressed proteins identified by LFQ. A significance score of 20% (equivalent to significance level of 0.01) and FDR ≤ 1% was considered statistically significant. In bioinformatics analysis, hypergeometric test followed with Benjamini and Hochberg FDR correction at *p* value < 0.05 (BiNGO built-in statistical tool) was used to correlate the association between functional annotation of genes and interacting proteins; the built-in statistical tool of KAAS was used to assess the possible association of interacting proteins and systemic pathways in the KEGG PATHWAY Database.

## 3. Results

### 3.1. OSLP Safety Study in Adult Zebrafish

#### 3.1.1. Behavioral Study

##### Swim Path Analysis

**Figure 2:**
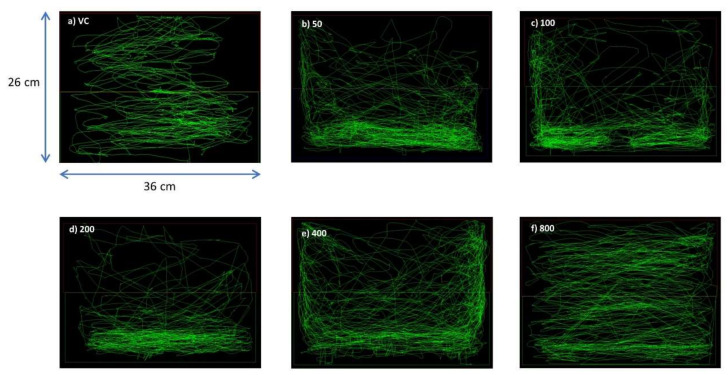
Representative swim paths for the corresponding 6 experimental groups (*n* = 8). (**a**) VC (tank water only, i.p.), (**b**) OSLP (50 μg/kg, i.p.), (**c**) OSLP (100 μg/kg, i.p.), (**d**) OSLP (200 μg/kg, i.p.), (**e**) OSLP (400 μg/kg, i.p.) and (**f**) OSLP (800 μg/kg, i.p.).

As seen in the swim paths generated by PANLAB SMART v3.0 software, the VC group (Figure 2a) swam throughout the whole tank without showing apparent preference for any part of the tank. The OSLP-treated groups, 50, 100, 200, and 400 µg respectively, showed slight preferences for the bottom half of tank when compared to the VC group (Figure 2b–e). In comparison, the OSLP 800 µg group (Figure 2f) showed a similar swimming pattern to the VC group as they swam throughout the whole tank with no apparent preference for any part of the tank. The 1600 μg/kg dose was excluded for causing mortality after an extended duration.

#### 3.1.2. Locomotion Parameters

**Figure 3:**
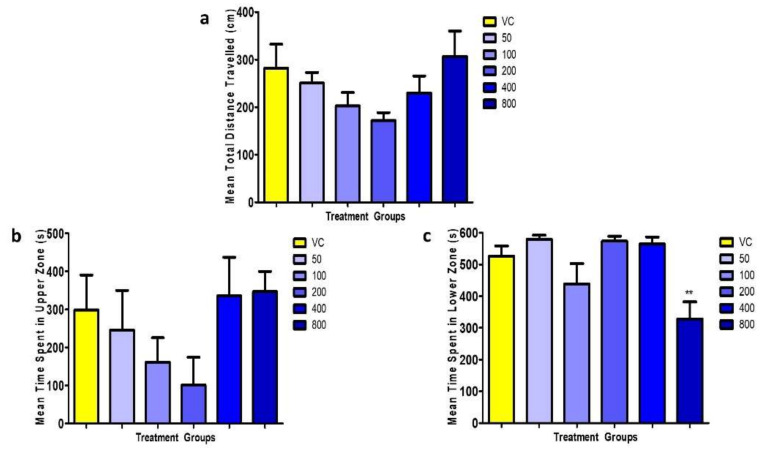
Mean locomotion parameters over 600 s for all the experimental groups. Figure (**a**) represents the mean total distance travelled (cm), Figure (**b**) shows the mean time spent in upper zone (s) and Figure (**c**) displays the mean time spent in lower zone (s). The data are expressed as Mean ± SEM, *n* = 8 and was analyzed using One-way ANOVA followed with Dunnett’s post-hoc test at significance level of ** *p* < 0.01 against the VC group (tank water only, i.p.).

For the mean total distance travelled, no significant differences (F = 1.798, *p* > 0.05) were found between the untreated VC group and all the OSLP-treated groups (50–800 µg/kg) (Figure 3a).

For another locomotion parameter, time spent in upper half of tank (s), no significant differences (F = 1.408, *p* > 0.05) were found between the untreated VC group and all the OSLP-treated groups Figure 3b, c). In a similar trend, no significant differences were also found in the mean time spent in lower half of tank, except in the OSLP 800 µg group. The zebrafish treated with OSLP 800 µg/kg spent a shorter time in the lower half of tank, 328 ± 54 s (F = 6.596, ** *p* < 0.01) than the VC group.

Based on the swim path analysis (Figure 2) and locomotion parameters (Figure 3), OSLP ranging from 50 to 800 µg did not result in any abnormal behavioral changes in the adult zebrafish. These doses were found to be safe for the use in adult zebrafish and did not result in any mortality or morbidity. Considering the maximum protective effects OSLP could possibly exhibit in brain at a safe concentration, 800 µg was fixed as the maximum safe starting dose. Subsequently, OSLP at 800 µg was used as the treatment dose across all further studies in this work. On the other hand, mortality in the adult zebrafish was recorded when treated with 1600 µg of OSLP. Therefore, this dose was considered as unsafe and the findings were not included in this work.

### 3.2. Evaluation of Anticonvulsive Potential of OSLP

#### 3.2.1. Behavioral Study

##### Swim Path Analysis

**Figure 4:**
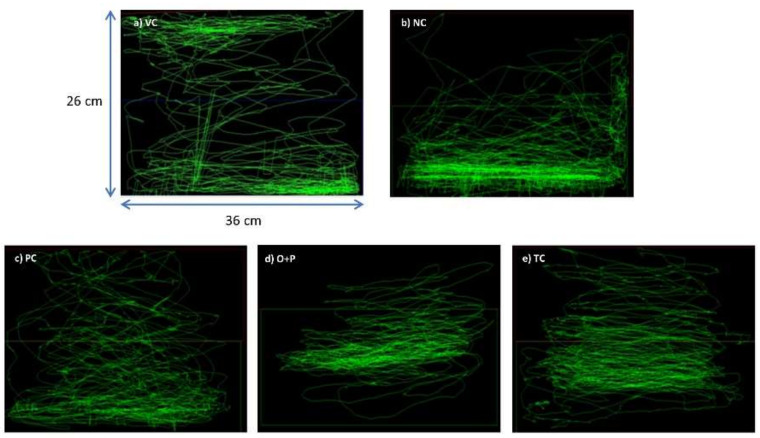
Representative swimming patterns for the corresponding 5 experimental groups (*n* = 10). VC ((**a**) tank water, i.p.), NC ((**b**) PTZ 170 mg/kg, i.p.), PC ((**c**) DZP 1.25 mg/kg + PTZ 170 mg/kg, i.p.), O+P ((**d**) OSLP 800 µg/kg + PTZ 170 mg/kg, i.p.) and TC ((**e**) OSLP 800 µg/kg + tank water, i.p.).

The VC group (Figure 4a) managed to swim throughout the entire tank without showing apparent preference for any part of the tank. In contrast, the NC group showed a more erratic swimming pattern after the PTZ challenge, with the zebrafish dwelling at the bottom half of tank more frequently (Figure 4b). Pre-treatment with DZP (PC group) modified the post PTZ challenge swimming behavior into a swimming pattern comparable to the VC group, with roughly equal amount of time being spent at the top and bottom of the tank (Figure 4c). Pre-treatment with OSLP 800 µg (O+P) also produced a swimming pattern similar to that of the VC group, without showing apparent preference for any part of the tank (Figure 4d). The treatment control group (TC) managed to swim throughout the whole tank without showing erratic swimming pattern (Figure 4e).

#### 3.2.2. Seizure Score and Seizure Onset Time

The cutoff time for seizure scoring was 600 s as fish fully recovered from seizures by 600 s. Mean seizure onset time for both the VC group and TC group were set as 600 s and a maximum seizure score of zero was assigned to these groups. They did not receive any PTZ challenges and thus did not show seizures. They served only as the study controls. PTZ injection into the zebrafish resulted in diverse seizure profiles, intensities, and latency in reaching the different seizure scores and onset time.

The NC group injected with PTZ had a significant increase in seizure score to 2.8 (F = 34.35 *** *p* < 0.001) and had significantly prompted the seizure onset time to the lowest, 76 s (F = 49.50, *** *p* < 0.001), when compared to the VC group. Higher seizure score with a concurrent lower seizure onset time indicated more severe seizures in the PTZ-injected zebrafish. Treatment with 800 µg/kg of OSLP showed a significant decrease in seizure score, to 1.5 (F = 34.35, *** *p* < 0.001) and significantly delayed the seizure onset time to 349 s (F = 49.50, *** *p* < 0.001) compared to the NC group. As expected, the PC group treated with DZP also showed a significant decrease in seizure score, to < 1 (F = 34.35, *** *p* < 0.001) and had significantly delayed the seizure onset time to 564 s (F = 49.50, *** *p* < 0.001) compared to the NC group.

**Figure 5:**
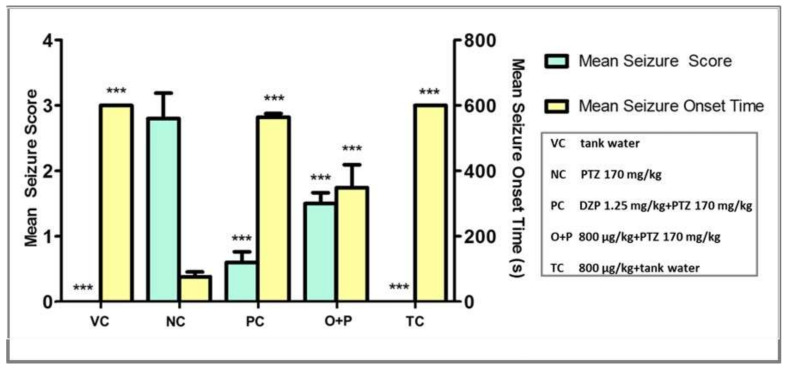
Mean seizure scores and mean seizure onset time (s) for the corresponding 5 experimental groups. Data are mean ± SEM. Experiments were repeated in *n* = 10, *** showed *p* < 0.001 against negative control. One-way ANOVA with Dunnett’s post-hoc test. VC (tank water, i.p.), NC (PTZ 170 mg/kg, i.p.), PC (DZP 1.25 mg/kg + PTZ 170 mg/kg, i.p.), O+P (OSLP 800 µg/kg + PTZ 170 mg/kg, i.p.) and TC (OSLP 800 µg/kg + tank water, i.p.).

In this study, PTZ (170 mg/kg of b.w.) was shown to sufficiently induce seizures in the adult zebrafish, with a high seizure score and a fast seizure onset time. OSLP treatment (800 µg/kg of b.w.) was shown to reduce seizure severity, with a lower seizure score and delayed the onset time to the most serious seizure score 4 (Figure 5).

#### 3.2.3. Locomotion Parameters

**Figure 6:**
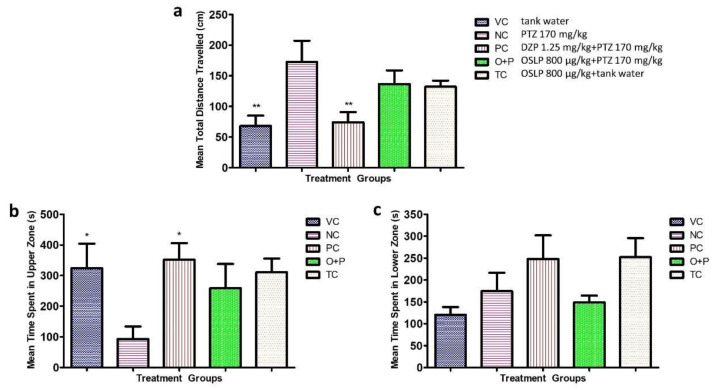
Mean locomotion parameters over 600 s for all the experimental groups. Figure (**a**) represents the mean total distance travelled (cm), Figure (**b**) shows the mean time spent in upper zone (s) and Figure (**c**) displays the mean time spent in lower zone (s). The data are expressed as Mean ± SEM, *n* = 10 and was analyzed using One-way ANOVA followed with Dunnett’s post-hoc test at significance level of * *p* < 0.05 and ** *p* < 0.01 against the negative control group (NC, PTZ 170 mg/kg). VC (tank water, i.p.), PC (DZP 1.25 mg/kg + PTZ 170 mg/kg, i.p.), O+P (OSLP 800 µg/kg + PTZ 170 mg/kg, i.p.) and TC (OSLP 800 µg/kg + tank water, i.p.).

Mean total distance travelled of the VC group was 68±16 cm (F = 4.527, ** *p* < 0.01) and was significantly shorter than the NC group injected with PTZ (172 ± 34 cm, F = 4.527, **p < 0.01). The NC group travelled about a 60% longer distance than the VC group (Figure 6a). The PC group treated with DZP had mean total distance travelled of 74 ± 16 cm which was 57% shorter than the PTZ-injected group (F = 4.527, ** *p* < 0.01) (Figure 6a). This significant improvement was also comparable to the VC group (68 ± 16 cm). A reduction was also seen in the O+P group, with mean total distance travelled of 137 ± 22 cm, which was about 20% shorter than the PTZ-induced alone group (Figure 6A).

For the parameter of time spent in each half of the tank, only groups VC and PC showed a significant longer time spent in the upper half of tank (324 ± 80 s and 352 ± 54 s respectively, F = 2.716, * *p* < 0.05) and a visibly shorter time spent in the bottom of tank than the NC group (Figure 6b,c). The O+P group (Figure 6b) had a trend of spending a slightly longer time in the upper half of tank (259 ± 78 s) but a slightly shorter time in the bottom half (149 ± 15 s) compared to the NC group (Figure 6c).

It is also worthy to mention that the TC group had displayed a similar trend to the VC group in all three locomotion parameters (Figure 6a–c). OSLP at a dose of 800 µg/kg did not trigger any locomotor manipulations and hence, was considerably safe in the adult zebrafish (Figure 6a–c).

#### 3.2.4. Neurotransmitter Study

**Figure 7:**
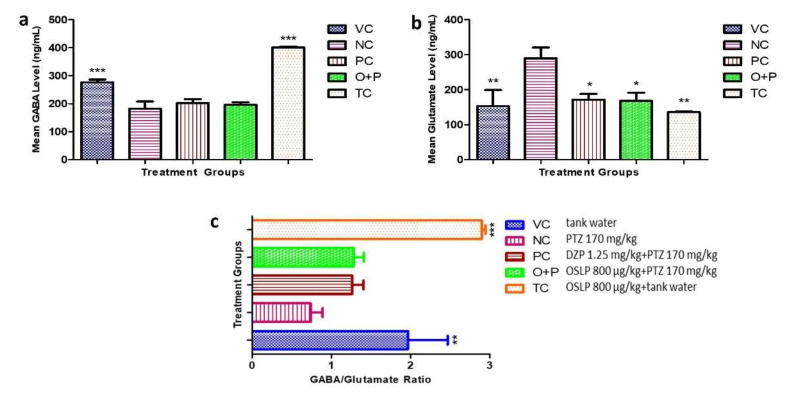
Mean neurotransmitter levels (ng/mL), namely GABA (**a**), glutamate (**b**) and GABA/Glu ratio (**c**) over 600 s for all the experimental groups. The data are expressed as Mean ± SEM, *n* = 10 and was analyzed using One-way ANOVA followed with Dunnett’s post-hoc test at significance level of * *p* < 0.05, ** *p* < 0.01 and *** *p* < 0.001 against the negative control group (NC, PTZ 170 mg/kg). VC (tank water, i.p.), PC (DZP 1.25 mg/kg + PTZ 170 mg/kg, i.p.), O+P (OSLP 800 µg/kg + PTZ 170 mg/kg, i.p.) and TC (OSLP 800 µg/kg + tank water, i.p.).

Neurotransmitters in the zebrafish brains, namely GABA and glutamate (Glu) and their ratio (GABA/Glu) were evaluated (Figure 7a–c).

The NC group showed a significant decrease in mean GABA levels (182 ± 26 ng/mL, *** *p* < 0.001) when compared to the VC group (276 ± 10 ng/mL, F = 37.74, *** *p* < 0.001) (Figure 7a). Mean GABA levels of the O+P group was 196 ± 9 ng/mL. Despite attaining about 7% higher GABA levels than the NC group, this treatment however did not attain statistical significance (Figure 7a). DZP treatment also brought about a slight increase in the GABA levels, to 202 ± 14 ng/mL as compared to 182 ± 26 ng/mL in the NC group (*p* > 0.05) (Figure 7a). Similarly, both PC (202 ± 14 ng/mL) and O+P (196 ± 9 ng/mL) had showed just slightly higher GABA levels than the NC group (Figure 7a).

In contrast, NC group showed a significant increase in Glu level (290 ± 30 ng/mL, F = 4.779, ** *p* < 0.01) when compared to the VC and TC groups (153 ± 46 ng/mL and 136 ± 2 ng/mL respectively, F = 4.779, ** *p* < 0.01) (Figure 7b). Meanwhile, DZP treatment brought about a significant decrease to 172 ± 16 ng/mL in the Glu levels, or about 41% lower than the NC group (F = 4.779, * *p* < 0.05) (Figure 7b). Mean Glu levels of the O+P group was 169 ± 22 ng/mL, which was 42% significantly lower than the NC group (F = 4.779, * *p* < 0.05) (Figure 7b). This outcome thus suggests that OSLP treatment could be effectively lower the Glu concentrations, normalizing it to the level comparable to those treated with DZP.

In addition, PTZ injection caused a significant decrease in the GABA/Glu ratio to <1, whereas, both VC and TC groups had a higher GABA/Glu ratio (R > 2, F = 13.81, ** *p* < 0.01 and *** *p* < 0.001, respectively) (Figure 7c). Very noteworthy is that the OSLP-treated and DZP-treated groups had a similar GABA/Glu ratio, though they did not attain statistical significance when compared to the NC (Figure 7c). It is worth mentioning that the TC group (treatment dose control) had higher GABA levels (401 ± 3 ng/mL, F = 37.74, *** *p* < 0.001) (Figure 7a) and lower Glu levels (136 ± 2 ng/mL, F = 4.779, *** *p* < 0.001) (Figure 7b) than the VC group. Also, the GABA/Glu ratio was comparable to the VC group.

#### 3.2.5. Proteins Expression Profiling Using Mass Spectrometry-Based Label-Free Proteomic Quantification (LFQ)

**Figure 8:**
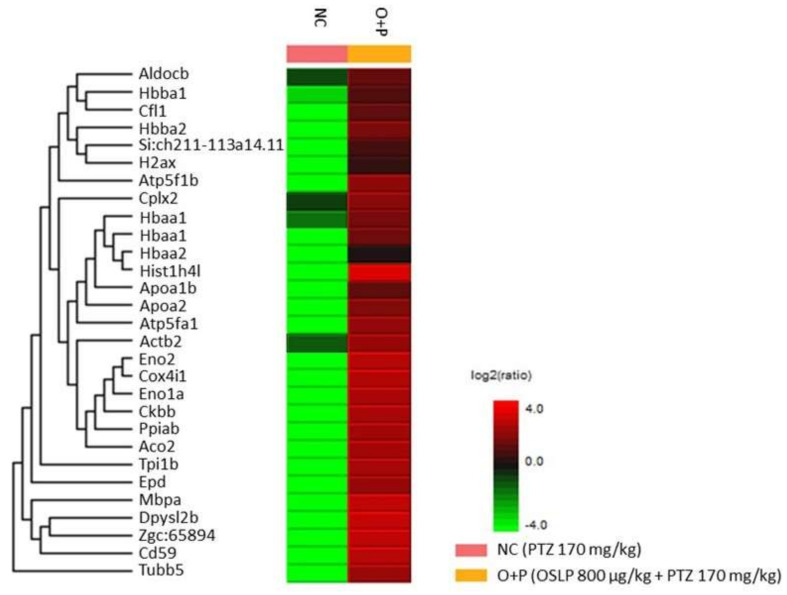
Heat map shows the differentially expressed proteins identified from negative control (NC, PTZ 170 mg/kg only) and O+P (OSLP 800 µg/kg + PTZ 170 mg/kg) zebrafish brains, *n* = 4, significance ≥ 20, FDR ≤ 1%, fold change ≥ 1, unique peptide ≥ 1. Protein names are listed on the left while experimental groups are indicated on top. The color key on the bottom right indicates the log2 (ratio) expression levels (green = low and red = high).

LFQ profiled 29 differentially expressed proteins from the brain samples of PTZ injected zebrafish (NC group) and the OSLP-treated PTZ group (O+P). These proteins were found to be expressed at lower levels in the NC group than in the O+P group (Figure 8 and Table 4). Among them, five proteins, namely hemoglobin subunit alpha (Hbaa1, isoforms Q803Z5 and Q90487), hemoglobin subunit beta-1 (Hbba1, Q90486), fructose-bisphosphate aldolase C-B (Aldocb, Q8JH70), actin beta 2 (Actb2, A8WG05), and complexin 2 (Cplx2, E7FBR8) were found expressed at higher levels.

#### 3.2.6. Bioinformatics Analysis

The differentially expressed proteins (Table 4) were searched in the ZFIN Database Information to match the gene ID. The database of InterPro Classification of Protein Families was searched for the respective protein class. The results were presented in Table 5.

**Figure 9:**
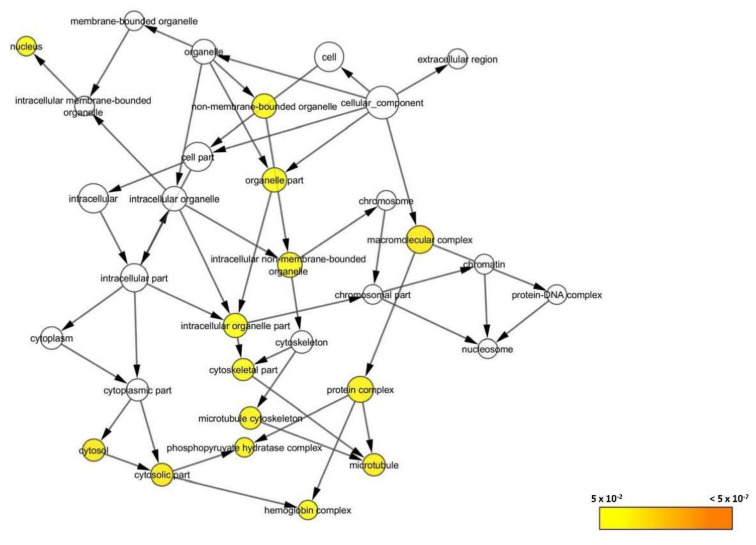
BiNGO result for cellular component as visualized in Cytoscape (Organism: *Danio rerio*). Colored nodes are significantly overrepresented. White nodes are not significantly overrepresented; they are included to show the colored nodes in the context of the GO hierarchy. Color key on the bottom right indicates the significance level of overrepresentation.

In addition, the differentially expressed proteins were found to localize at 14 different cellular components; mainly at the organelle part (GO:44422) and the macromolecular complex (GO:32991). They were localized at the intracellular non-membrane-bounded organelle (GO:43232), intracellular organelle part (GO:44446), cytoskeletal part (GO:44430), microtubule cytoskeleton (GO:15630), microtubule (GO:5874), protein complex (GO:43234), phosphopyruvate hydratase complex (GO:15), hemoglobin complex (GO:5833), cytosol (GO:5829), cytosolic part (GO:44445), and non-membrane-bounded organelle (GO:43228). The identified proteins were also localized at the nucleus of the cell part (GO:5634) (Figure 9).

**Figure 10:**
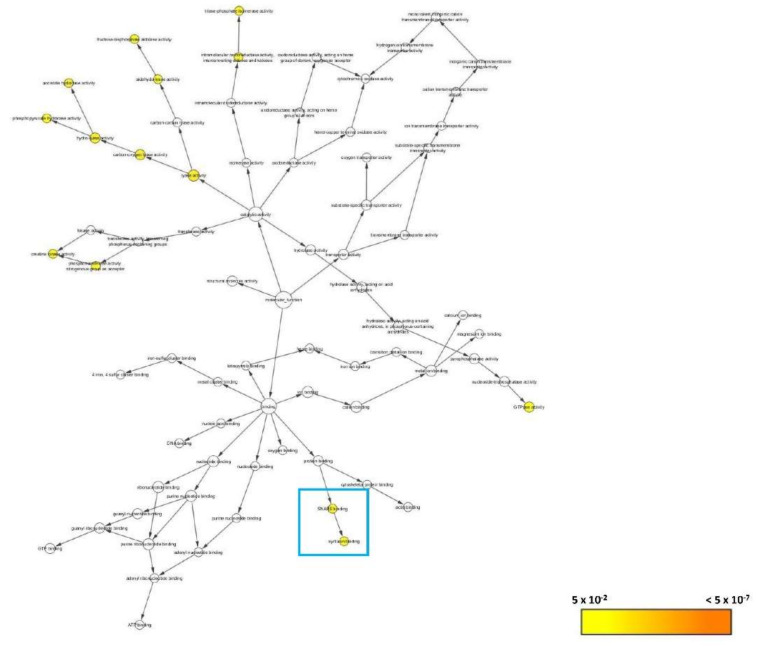
BiNGO result for molecular function as visualized in Cytoscape (Organism: *Danio rerio*). Colored nodes are significantly overrepresented. White nodes are not significantly overrepresented; they are included to show the colored nodes in the context of the GO hierarchy. Color key on the bottom right indicates the significance level of overrepresentation.

The proteins that were found localized at the different cellular components aforementioned have their interactions significantly associated with 14 corresponding molecular functions (Figure 10). Around 12 were involved in catalytic activities of lyase (GO:16829), carbon-oxygen lyase (GO:16835), hydro-lyase (GO:16836), phosphopyruvate hydratase (GO:4634), aconitate hydratase (GO:3994), fructose-bisphosphate aldolase (GO:4332), and aldehyde-lyase (GO:16832); triose-phosphate isomerase (GO:4807), and intramolecular oxidoreductase which interconverts aldoses and ketoses (GO:16861); creatine kinase (GO:4111) and phosphotransferase uses nitrogenous group as acceptor (GO:16775), and GTPase activity (GO:3924). Two protein interactions were particularly noteworthy: the bindings of SNARE (GO:149) and syntaxin (GO:19905) (blue box; Figure 10).

#### 3.2.7. Systematic Pathway Enrichment Analysis

KEGG PATHWAY database (Organism: *Danio rerio*) revealed that the differentially expressed proteins were significantly associated with six major categories of pathways; five of them were associated with metabolism, genetic information processing, environmental information processing, cellular processes, and organismal systems whilst the last one was associated with human diseases (see Table 6).

**Figure 11:**
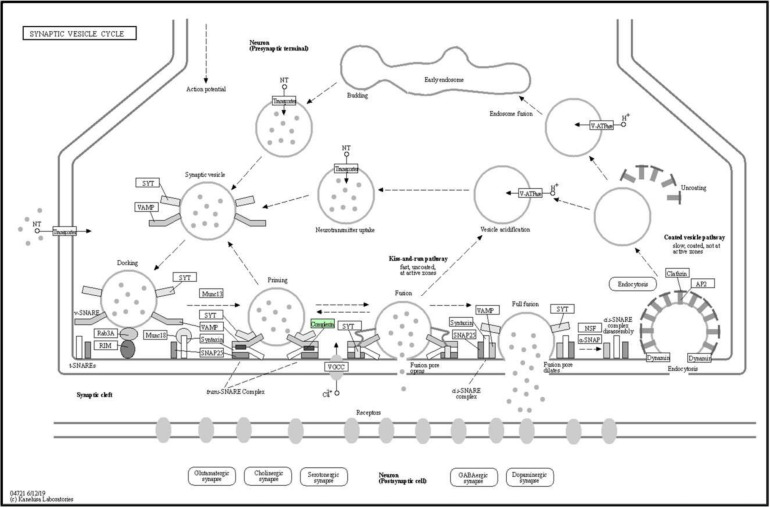
Complexin 2 in green box was mapped onto the synaptic vesicle cycle pathway (04721) generated by KEGG PATHWAY (Organism: *Danio rerio*). Solid arrows represent molecular interactions or relations whereas dashed arrows represent indirect links or unknown reactions.

The synaptic vesicle cycle (04721) in the nervous system that is nested under the organismal systems category, was the pathway most likely to play a significant role (Table 6). Complexin 2 (Cplx2) was mapped onto the synaptic vesicle cycle pathway (04721), highlighted in a green box as Complexin (Figure 11). Cplx2 is an isoform of the complexin protein family with four isoforms, Cplx1–4. As shown in a synapse, during priming phase, Cplx2 is bound to the trans-SNARE complex together with synaptotagmin (Syt), vesicle-associated membrane protein (Vamp), syntaxin (Stx), and synaptosomal-associated protein of 25 kDa (Snap25). SNARE binding of complexin is essential for normal priming at the presynaptic plasma membrane, which is known as the active zone, and subsequent Ca^2+^-evoked neurotransmitter release.

## 4. Discussion

This study investigates the maximum safe starting dose of OSLP and elucidates its anticonvulsive potential in PTZ-induced adult zebrafish seizures.

Firstly, the maximum safe starting dose of OSLP to be used for anticonvulsive activity determination in the adult zebrafish [14,18] was evaluated. In this study, OSLP concentrations ranging from 50–1600 µg/kg of b.w. were tested in each assigned group. The zebrafish swimming pattern after exposure to 800 µg of OSLP did not show bottom-dwelling behavior. Diving to the bottom of tank can be a natural reflexive response of zebrafish. However, increased bottom-dwelling behavior has been linked to anxiety in the novel tank test [15,16]. The bottom dwelling frequency has been found reduced in zebrafish when treated with anxiolytic compounds [7,17]. These earlier findings thus lend support to the anxiolytic potential of OSLP in adult zebrafish, at least at a concentration greater than 800 µg/kg of body weight. Noteworthy however, OSLP at a concentration of 1600 µg is capable of causing lethal events in adult zebrafish. This finding has drawn a line to limit the maximum safe dose of OSLP achievable via intraperitoneal route to be not greater than 1600 µg/kg of body weight, at least in the case of zebrafish. This also lends support to the exclusion of 1600 µg OSLP for further analysis in this work. Building on the safety study outcomes and considering the maximum protective effects of OSLP at a safe concentration, 800 µg was chosen as the treatment dose in this study.

The OSLP safety study was crucial as there was no prior published scientific evidence on OSLP in both in vitro and in vivo models, let alone its neuroprotective potential. A prior literature search only yielded two studies on the ethanolic extracts of *Orthosiphon stamineus*; Choo et al. (2018) examined the anticonvulsive potential in adult zebrafish [9] and Ismail et al. (2017) reported on toxicity in zebrafish embryos [19]. As such, this work represents the first of its kind.

In this study, the PTZ-induced seizure model was established [7,12] to investigate the anticonvulsive potential of OSLP (800 µg/kg of b.w.) using adult zebrafish. Pre-treatment with OSLP 800 µg for 30 min brought about significant improvements in the PTZ-injected zebrafish, with a lower seizure score and a prolonged seizure onset time. Pre-treatment with OSLP 800 µg also produced a swimming pattern comparable to that of the untreated VC which received neither PTZ injection nor OSLP treatment (TC). It was seen that the O+P group managed to swim through the whole tank without showing an apparent preference for any spot or apparent bottom-dwelling behavior. Contradictorily, the representative zebrafish swimming pattern showed a bottom-dwelling behavior in the PTZ-injected group, which has been strongly linked to the anxious behavior in seizures [15,16]. A similar observation was also reported in two recent studies using PTZ-induced zebrafish [7,12]. Diazepam (DZP, 1.25 mg/kg) has been found in this study to efficaciously control seizures in the PTZ-injected zebrafish and thus, a swimming pattern comparable to that of the untreated VC was observed. Interestingly, the TC group which received neither PTZ injection nor DZP treatment, produced a swimming pattern comparable to that of the untreated VC group. This finding thus reaffirms that OSLP at 800 µg/kg of body weight does not produce lethal events and with that it could be potentially anticonvulsive. Nevertheless, one of the limitations in this study includes a considerably low yield of OSLP (approximately 0.3%) extracted from OS leaves and hence, based on the safety study (Section 3.1), only the maximal safe dose (800 µg/kg of b.w.) was used.

The PTZ-injected group had the highest mean total distance travelled and travelled about 60% longer distance than the untreated VC group. This uncontrolled movement has been strongly linked to burst neuronal firing in addition to the pass-out phenomenon in seizures [20,21]. A similar observation was also reported in two recent studies using PTZ-induced zebrafish [7,12]. A disruption occurred in the normal balance of excitation and inhibition following the injection of PTZ. Binding of PTZ to GABA_A_ (γ-aminobutyric acid type A) receptors stimulated excitability in the brains and hence provoked uncontrolled seizures in the zebrafish. This explains the representative swim path of the PTZ-injected group which showed burst swimming activities (i.e., erratic movements, loss of direction) which taken together, contributed to the longest total distance travelled. Moreover, the PTZ-injected group spent a longer time in the lower half of tank, which could possibly be attributed to the bottom-dwelling behavior in seizures [15,16].

In contrast, pre-treatment with DZP significantly alleviated the manipulations of PTZ. A 57% reduction in the total distance travelled was seen in the DZP-treated group and it spent more time in the upper half of tank in a comparable manner to that of the untreated group. Interestingly however, it also spent a longer time in the bottom half of the tank, but the untreated group did not. This phenomenon could be attributed to the sedative effects of DZP. DZP is an anxiolytic benzodiazepine with fast-acting and long-lasting actions [22]. When administered intravenously, DZP has been shown to act within 1 to 3 min, while oral dosing onset ranges between 15 to 60 min; with a duration of action of more than 12 h. Similar to most benzodiazepines, DZP causes adverse effects including syncope (temporary loss of consciousness), sedation and confusion, to name a few [23]. A similar finding was also reported in three studies using DZP to treat zebrafish [7,12,24]. Pre-treatment with OSLP 800 µg also alleviated the manipulations of PTZ. A 20% reduction in the total distance travelled was seen in the OSLP-treated group and similarly, they spent more time in the upper half of tank compared to the DZP-treated group. Interestingly however, they did not spend a longer time in the bottom half of tank as the DZP-treated group did, but in a pattern more comparable to the untreated VC group. Hence, this outcome suggests that OSLP’s anticonvulsive actions could be acting differently from DZP and with that, it might not produce the similar cognitive insults such as DZP. This similar outcome has been reported in Choo’s study using *O. stamineus* ethanolic extracts to treat adult zebrafish [7]. On the market, DZP has since been one of the top selling AEDs of all time, well known for its fast onset of action and is often effective in adults [25,26]. However, DZP’s high clinical efficacy in treating epilepsy and seizures comes with multiple adverse reactions such as suicidality, paradoxical CNS stimulation, syncope, sedation, depression and dystonia, to name a few [23]. These adverse effects are common in currently available AEDs. Worthy of mention, the TC group did not show any abnormal locomotion parameters and hence, reaffirming that this dose is considerably safe in the adult zebrafish.

Taken together, the outcomes of behavioral study suggest that OSLP at 800 µg/kg of body weight is potentially anticonvulsive. OSLP treatment produced milder anticonvulsant effects in comparison to DZP treatment, which is one of the standard AEDs available today.

In this study, two major neurotransmitters, namely GABA and Glu, were investigated. An interrupted GABA/Glu cycle was seen in the PTZ-injected zebrafish, with a drop in the mean GABA level but a surge in the mean Glu level. Distinctively, such anomalies were not found in the untreated zebrafish which did not receive PTZ injection. Additionally, the GABA/Glu ratio of PTZ-injected group remained the lowest. This thus shows a disruption in the normal balance of excitation and inhibition following the PTZ treatment. PTZ is a tetrazol derivative known to block GABA_A_ receptor function [27]. PTZ suppresses GABA inhibitory activities which in turn potentiates the Glu excitatory activities in the brain and eventually results in an unbalanced GABA/Glu ratio. This finding has lent more support to the severe seizures seen in the PTZ-injected group. Pre-treatment with DZP, without surprise, significantly suppressed the excitatory neurotransmitter Glu, normalizing it to be comparable to the untreated VC group. Concurrently, the GABA levels in the DZP-treated group saw a slight elevation and this eventually improved the GABA/Glu ratio. A similar finding has been reported earlier [28]. DZP inhibits Glu release to suppress glutamatergic hyperactivity and hence, restores the balance between GABA and Glu to promptly arrest neuroexcitation [29,30]. Pre-treatment with OSLP has also improved the neurotransmitters profile, with significantly lower excitatory Glu levels. More interestingly, OSLP treatment brings the GABA/Glu ratio close to the DZP treatment. Although to a lesser degree than the pure drug control, taken together, these findings show that OSLP has GABA potentiating actions and antiglutamatergic effects. Moreover, the finding that TC group had a neurotransmitters profile comparable to the untreated VC group, has also buttressed the proposal of OSLP could be having neuroprotective potential.

The present protein expression study is useful in helping to predict the anticonvulsive mechanism of OSLP. The main findings are the following. First, mass spectrometry-based LFQ analysis compared the differentially expressed proteins in the seizure group (NC, induced by PTZ 170 mg/kg only) and the OSLP-treated seizure group (OSLP 800 µg/kg + PTZ 170 mg/kg). This identified a distinct protein expression profile of 29 differentially expressed proteins that had higher expressions in the O+P group than in the NC group. Second, functional annotation analysis found the protein bindings of SNARE (GO:149) and syntaxin (GO:19905) at intracellular localizations that were particularly interesting, given the fundamental role they play in the regulation of membrane fusion during presynaptic vesicle exocytosis. Third, KEGG pathway mapping proposed the synaptic vesicle cycle (04721) as the most probable pathway, in line with the strong association between SNARE and syntaxin proteins. These proteins are required in calcium (Ca^2+^)-dependent synaptic vesicle exocytosis. As shown, the trans-SNARE complex was assembled in the presence of SNARE proteins including complexin (Cplx), syntaxin (Stx), synaptotagmin (Syt), synaptosomal-associated protein of 25 kDa (Snap25) and vesicle-associated membrane protein (Vamp). According to ZFIN (https://zfin.org/ZDB-GENE-081113-1), gene cplx2 is predicted to orthologous to human gene CPLX2.

Complexin is an important regulator of synaptic vesicle exocytosis. Complexins, also called synaphins, are small cytosolic proteins. They form a small protein family with four isoforms, Cplx1–4 [31]. Cplx1 and Cplx2 are highly homologous. In particular, they bind to the SNARE complex which are expressed at presynaptic sites [32,33,34,35]. SNARE binding is a highly specialized regulation that is strictly regulated by synaptic fusion machinery. The basic components of a synaptic fusion machinery are the SNARE proteins namely Cplx, Stx, Syt, Snap25, Vamp, and two mammalian uncoordinated proteins (Munc13 and Munc18) [36]. The formation of the trans-SNARE complex is required in the vesicle priming phase. As the trans-SNARE complex forms, the vesicle is pulled close to the plasma membrane, where it is ready to fuse in response to the Ca^2+^ influx that is triggered by an action potential, usually in less than a millisecond. Complexin binds to the trans-SNARE complex and modulates the fusion process by either increasing or decreasing the height of the energy barrier for fusion. The height of the energy barrier for fusion is not only important for evoked release but also determines how likely vesicles are to fuse spontaneously in the absence of a Ca^2+^-triggering signal. After fusion, the vesicle is retrieved by endocytosis and reloaded for another round of exocytosis [13,32,33,36]. Therefore, the binding of complexin to the SNARE complex is crucial for the normal priming and subsequent Ca^2+^-evoked neurotransmitter release during presynaptic vesicle exocytosis.

The findings of protein expression study have suggested that synaptic vesicle cycle pathway could play a significant role in modulating the anticonvulsive mechanism of OSLP. OSLP could be regulating the release of GABA and Glu via calcium-dependent synaptic vesicle exocytosis. Similar findings have been reported by studies using samples from rats and patients [32,34,35]. Decreased expressions of complexin 2 have also been associated with neurodegenerative diseases including Alzheimer’s, Huntington’s, and Parkinson’s; psychiatric disorders including schizophrenia and bipolar disorder [37,38,39,40], with seizures and epilepsy being common comorbidities [41,42,43,44,45,46].

OSLP could be a potential anticonvulsant. Found in OSLP, baicalein 7-*O*-glucuronosyltransferase and baicalin-beta-D-glucuronidase are responsible for the biosynthesis of baicalein and baicalin, respectively. Baicalein and baicalin have been reported to have anxiolytic activity and acting on GABA and glutamic acid in rat brains [47], binding to the benzodiazepine site of the GABA_A_ receptor to potentiate GABA-mediated inhibition [48,49,50] and anticonvulsive action in the PTZ-induced seizure rat model [51]. Beta-mycrene synthase and R-linalool synthase are proteins responsible for the biosynthesis of myrcene and linalool respectively. Linalool has been reported to have antiepileptiform and antiseizure properties in PTZ-treated rats [52,53,54] whereas beta-mycrene has also been reported for sedative effects in human [55] and anticonvulsive effects in PTZ-treated rats [56]. Beta-mycrene synthase and R-linalool synthase might not directly act on cannabinoid receptors but could be producing synergic effects with future cannabinoid-based AEDs. The postulated synergistic contribution on both GABA and Glu neurotransmitters can increase the efficacy of future cannabinoid-based AEDs in managing epilepsy and seizures [57,58,59,60]. Rosmarinate synthase is involved in the biosynthesis of rosmarinic acid Choo, Kundap [7] suggested that rosmarinic acid (in an ethanolic extract of OS) is one of the probable antiepileptic components of the extract in adult zebrafish whereas similar findings in PTZ-induced seizures in mice have also been reported earlier [10,61].

## 5. Conclusions

The study suggests that OSLP could be a potential anticonvulsant. OSLP most likely regulates the release of the neurotransmitters, GABA and Glu, via calcium-dependent synaptic vesicle exocytosis mediated by the “synaptic vesicle cycle” pathway. To the best of our knowledge, this study is the first to show that OSLP can safely ameliorate epilepticeizures in adult zebrafish.

## Figures and Tables

**Table 1:** Experimental groups in OSLP safety study.

Group	Treatment
VC	Vehicle control (tank water, i.p.)
Treatment Group a	OSLP (50 µg/kg, i.p.)
Treatment Group b	OSLP (100 µg/kg, i.p.)
Treatment Group c	OSLP (200 µg/kg, i.p.)
Treatment Group d	OSLP (400 µg/kg, i.p.)
Treatment Group e	OSLP (800 µg/kg, i.p.)
Treatment Group f	OSLP (1600 µg/kg, i.p.)

**Table 2:** Experimental groups in the evaluation of OSLP anti-convulsive potential.

Group	Treatment
VC	Vehicle control (tank water, i.p. + tank water)
NC	Negative control (tank water + PTZ 170 mg/kg, i.p.)
PC	Positive control (DZP 1.25 mg/kg + PTZ 170 mg/kg, i.p.)
TC	Treatment control (800 µg/kg + tank water, i.p.)
O+P	OSLP-treated PTZ (800 µg/kg + PTZ 170 mg/kg, i.p.)

**Table 3:** Seizure scoring system.

Score	Criteria
1	short swim mainly at the bottom of tank
2	increased swimming activity and high frequency of opercular movements
3	burst swimming, left and right movements as well as the erratic movements
4	circular movements

**Table 4:** Differentially expressed proteins identified from negative control (NC, PTZ 170 mg/kg only) and O+P (OSLP 800 µg/kg + PTZ 170 mg/kg) zebrafish brains.

Uniprot Accession ID	Uniprot Protein Name	Significance (≥13)	Coverage (%)	#Peptides	#Unique	Avg. Mass	Group Profile (Ratio of NC/O+P)	ZFIN Protein
Q90487	Hemoglobin subunit alpha	200	49	12	4	15,524	0.00:1.00	Hbaa1
Q803Z5	Hemoglobin subunit alpha	200	49	12	4	15,508	1.00:255.14	Hbaa1
Q90486	Hemoglobin subunit beta-1	200	43	8	8	16,389	1.00:49.49	Hbba1
Q08BA1	ATP synthase subunit alpha	200	15	6	3	59,744	0.00:1.00	Atp5fa1
Q6PC12	Enolase 1	200	12	4	3	47,074	0.00:1.00	Eno1a
Q4VBK0	ATP synthase subunit beta	200	9	3	3	55,000	0.00:1.00	Atp5f1b
Q6ZM12	Hemoglobin beta adult 2	200	13	3	3	16,295	0.00:1.00	Hbba2
E7F2M5	CD59 molecule (CD59 blood group)	200	26	2	2	12,914	0.00:1.00	Cd59
E9QBF0	Triosephosphate isomerase	200	20	4	4	21,811	0.00:1.00	Tpi1b
Q6PC53	Peptidyl-prolyl cis-trans isomerase	200	18	3	3	17,489	0.00:1.00	Ppiab
F8W4M7	Aconitate hydratase mitochondrial	200	4	2	2	85,590	0.00:1.00	Aco2
Q8AY63	Brain-subtype creatine kinase	153.53	7	3	3	42,884	0.00:1.00	Ckbb
Q4VBT9	Cox4i1 protein	126.93	18	2	2	19,443	0.00:1.00	Cox4i1
Q8JH70	Fructose-bisphosphate aldolase C-B	126.51	12	4	4	39,259	1.00:8.14	Aldocb
Q6PE34	Tubulin beta chain	125.32	31	12	3	49,635	0.00:1.00	Zgc:65894
A0A0R4IKF0	Apolipoprotein A-Ib	112	9	2	2	30,140	0.00:1.00	Apoa1b
F8W3W8	Myelin basic protein a	110.44	63	10	2	10,776	0.00:1.00	Mbpa
A3KPR4	Histone H4	104.16	24	2	2	11,367	0.00:1.00	Hist1h4l
B3DFP9	Apolipoprotein A-II	103.56	21	2	2	15,537	0.00:1.00	Apoa2
Q5BJC7	Haemoglobin alpha adult 2	79.54	17	3	2	15,403	0.00:1.00	Hbaa2
Q8AYC4	Tubulin beta chain	76.34	39	13	2	49,826	0.00:1.00	Tubb5
R4GE02	Si:ch211–113a14.11	56.58	21	4	2	27,149	0.00:1.00	Si:ch211–113a14.11
Q7ZUY3	Histone H2AX	56.36	34	4	2	15,001	0.00:1.00	H2ax
Q6TH32	Cofilin 1	44.76	13	2	2	18,771	0.00:1.00	Cfl1
A0A2R8Q2Z0	Ependymin	44.72	12	2	2	23,370	0.00:1.00	Epd
A8WG05	Actin beta 2	43.68	15	6	6	41,753	1.00:9.75	Actb2
E7FBR8	Complexin 2	43.27	24	2	2	15,094	1.00:5.64	Cplx2
A8DZ95	Dihydropyrimidinase-like 2b	43.27	6	2	2	58,285	0.00:1.00	Dpysl2b
Q6GQM9	Eno2 protein	36.01	7	2	1	46,841	0.00:1.00	Eno2

Remark: ZFIN protein nomenclatures were searched in the ZFIN Database Information (*www.zfin.org*) as accessed on 17/02/2020.

**Table 5:** Protein family of the differentially expressed proteins identified from negative control (NC, PTZ 170 mg/kg only) and O+P (OSLP 800 µg/kg + PTZ 170 mg/kg) zebrafish brains.

Protein Family	ZFIN Protein	ZFIN Gene ID
**Globin domain-containing protein**		
Belongs to the family of hemoglobin, alpha-type and to the subfamily of hemoglobin, pi	Hbaa1	ZDB-GENE-980526-79
	Hbaa2	ZDB-GENE-081104-38
Member of the hemoglobin, beta-type	Hbba1	ZDB-GENE-990415-18
	Hbba2	ZDB-GENE-040801-164
**Plasma protein**		
Member of the CD marker	Cd59	ZDB-GENE-030131-7871
Member of the apolipoprotein A/E	Apoa2	ZDB-GENE-030131-1046
Belong to the myelin basic protein	Mbpa	ZDB-GENE-030128-2
Member of the apolipoprotein A/E	Apoa1b	ZDB-GENE-050302-172
**Cytoskeletal protein**		
Member of the actin family	Actb2	ZDB-GENE-000329-3
Member of the beta tubulin	Tubb5	ZDB-GENE-031110-4
	Zgc:65894	ZDB-GENE-030131-7741
**Enzyme protein**		
***Transferase***		
Member of the ATP:guanido phosphotransferase protein	Ckbb	ZDB-GENE-020103-2
Member of the ATP synthase, F1 complex, beta subunit	Atp5f1b	ZDB-GENE-030131-124
Member of the mitochondrial F1-F0 ATP synthase subunit F	Atp5mf	ZDB-GENE-050309-87
Member of the ATP synthase, F1 complex, alpha subunit	Atp5fa1	ZDB-GENE-060201-1
***Isomerase***		
Member of the cyclophilin-type peptidyl-prolyl cis-trans isomerase	Ppiab	ZDB-GENE-030131-7459
Member of the triosephosphate isomerase	Tpi1b	ZDB-GENE-020416-4
***Lyase***		
Member of the fructose-bisphosphate aldolase, class-I	Aldocb	ZDB-GENE-030821-1
Member of the enolase	Eno1a	ZDB-GENE-030131-6048
***Citric acid cycle related protein***		
Belongs to the family of aconitase, mitochondrial-like	Aco2	ZDB-GENE-030131-1390
Member of the enolase	Eno2	ZDB-GENE-040704-27
**Histone protein**		
***Core Histone***		
Member of the histone H2A	H2ax	ZDB-GENE-040426-987
	Si:ch211-113a14.11	ZDB-GENE-121214-162
Member of the histone H4	Hist1h4l	ZDB-GENE-070927-10
**Intracellular protein-Ependymin**		
Member of the ependymin-related protein family (EPDRs)	Epd	ZDB-GENE-980526-111
**Cytosolic protein**		
Member of the complexin/synaphin family	Cplx2	ZDB-GENE-081113-1
**ADF-H domain-containing protein**		
Belongs to the family of ADF/Cofilin	Cfl1	ZDB-GENE-030131-215
**Transporter protein-Primary active transporter**		
Belongs to the cytochrome c oxidase subunit IV family and to the subfamily of cytochrome c oxidase subunit IV	Cox4i1	ZDB-GENE-030131-5175
**Amidohydro-rel domain-containing protein**		
Belongs to the hydantoinase/dihydropyrimidinase family and to the subfamily of dihydropyrimidinase-related protein 2	Dpysl2b	ZDB-GENE-031105-1

Remark: Protein Families and their respective functions were searched in the InterPro Classification of Protein Families and the ZFIN Database Information (https://www.ebi.ac.uk/interpro/protein/UniProt/ and www.zfin.org accessed on 17 February 2020).

**Table 6:** KEGG pathways (Organism: *Danio rerio*) associated with the differentially expressed proteins identified from negative control (NC, PTZ 170 mg/kg only) and O+P (OSLP 800 µg/kg + PTZ 170 mg/kg) zebrafish brains.

KEGG Pathway ID	Pathway Category	Mapped Protein
	**Metabolism**	
**01100**	**Metabolic pathways**	Ckbb, Aldocb, Aco2, Eno1a, Eno2, Tpi1b, Atp5fa1, Atp5f1b, Cox4i1
01110	Biosynthesis of secondary metabolites	Aldocb, Aco2, Eno1a, Eno2, Tpi1b
01120	Microbial metabolism in diverse environments	Aldocb, Aco2, Eno1a, Eno2, Tpi1b
01200	Carbon metabolism	Aldocb, Aco2, Eno1a, Eno2, Tpi1b
01210	2-Oxocarboxylic acid metabolism	Aco2
01230	Biosynthesis of amino acids	Aldocb, Aco2, Eno1a, Eno2, Tpi1b
	***Carbohydrate metabolism***	
00010	Glycolysis/Gluconeogenesis	Aldocb, Eno1a, Eno2, Tpi1b
00020	Citrate cycle (TCA cycle)	Aco2
00030	Pentose phosphate pathway	Aldocb
00051	Fructose and mannose metabolism	Aldocb, Tpi1b
00630	Glyoxylate and dicarboxylate metabolism	Aco2
00562	Inositol phosphate metabolism	Tpi1b
	***Energy metabolism***	
00190	Oxidative phosphorylation	Atp5fa1, Atp5f1b, Cox4i1
00710	Carbon fixation in photosynthetic organisms	Aldocb, Tpi1b
00720	Carbon fixation pathways in prokaryotes	Aco2
00680	Methane metabolism	Aldocb, Eno1a, Eno2, Ppiab
	***Amino acid metabolism***	
00330	Arginine and proline metabolism	Ckbb
	**Genetic Information Processing**	
	***Folding, sorting and degradation***	
03018	RNA degradation	Eno1a, Eno2
	**Environmental Information Processing**	
	***Signal transduction***	
04015	Rap1 signaling pathway	Actb2
04390	Hippo signaling pathway	Actb2
04391	Hippo signaling pathway - fly	Actb2
04066	HIF-1 signaling pathway	Aldocb, Eno1a, Eno2
	**Cellular Processes**	
	***Transport and catabolism***	
04145	Phagosome	Actb2, Tubb5, Zgc:65894
	***Cell growth and death***	
04210	Apoptosis	Actb2
04217	Necroptosis	Ppiab, H2ax
	***Cellular community-eukaryotes***	
04510	Focal adhesion	Actb2
04520	Adherens junction	Actb2
04530	Tight junction	Actb2
04540	Gap junction	Tubb5, Zgc:65894
	***Cell motility***	
04810	Regulation of actin cytoskeleton	Actb2, Cfl1
	**Organismal Systems**	
	***Immune system***	
04640	Hematopoietic cell lineage	Cd59
04610	Complement and coagulation cascades	Cd59
04611	Platelet activation	Actb2
04666	Fc gamma R-mediated phagocytosis	Cfl1
04670	Leukocyte transendothelial migration	Actb2
	***Endocrine system***	
04921	Oxytocin signaling pathway	Actb2
04919	Thyroid hormone signaling pathway	Actb2
	***Circulatory system***	
04260	Cardiac muscle contraction	Cox4i1
	***Digestive system***	
04971	Gastric acid secretion	Actb2
	***Nervous system***	
04721	Synaptic vesicle cycle	Cplx2
	***Sensory system***	
04745	Phototransduction - fly	Actb2
	***Development and regeneration***	
04360	Axon guidance	Cfl1, Dpysl2b
	***Environmental adaptation***	
04714	Thermogenesis	Atp5fa1, Atp5f1b, Cox4i1, Actb2
	**Human Diseases**	
	**Cancer: overview**	
05205	Proteoglycans in cancer	Actb2
05203	Viral carcinogenesis	Si:ch211-113a14.11, Hist1h4l
	***Cancer: specific types***	
05225	Hepatocellular carcinoma	Actb2
	**Immune disease**	
05322	Systemic lupus erythematosus	H2ax, Si:ch211-113a14.11, Hist1h4l
	**Neurodegenerative disease**	
05010	Alzheimer disease	Atp5fa1, Atp5f1b, Cox4i1, Tubb5
05012	Parkinson disease	Atp5fa1, Atp5f1b, Cox4i1
05016	Huntington disease	Atp5fa1, Atp5f1b, Cox4i1, Tubb5
	**Substance dependence**	
05034	Alcoholism	H2ax, Si:ch211-113a14.11, Hist1h4l
	**Cardiovascular disease**	
05418	Fluid shear stress and atherosclerosis	Actb2
05410	Hypertrophic cardiomyopathy (HCM)	Actb2
05412	Arrhythmogenic right ventricular cardiomyopathy (ARVC)	Actb2
05414	Dilated cardiomyopathy (DCM)	Actb2
05416	Viral myocarditis	Actb2
	**Endocrine and metabolic disease**	
04932	Non-alcoholic fatty liver disease (NAFLD)	Cox4i1
	**Infectious disease: bacterial**	
05110	Vibrio cholerae infection	Actb2
05130	Pathogenic Escherichia coli infection	Actb2, Tubb5, Zgc:65894
05132	Salmonella infection	Actb2
05131	Shigellosis	Actb2
05135	Yersinia infection	Actb2
05133	Pertussis	Cfl1
05100	Bacterial invasion of epithelial cells	Actb2, Cfl1
	**Infectious disease: viral**	
05164	Influenza A	Actb2
	**Drug resistance: antimicrobial**	
01503	Cationic antimicrobial peptide (CAMP) resistance	Ppiab
